# Incidence of Active Pulmonary Tuberculosis in Patients with Coincident Filarial and/or Intestinal Helminth Infections Followed Longitudinally in South India

**DOI:** 10.1371/journal.pone.0094603

**Published:** 2014-04-11

**Authors:** Soumya Chatterjee, Chockalingam Kolappan, Rangasamy Subramani, Punnathanathu G. Gopi, Vedhachalam Chandrasekaran, Michael P. Fay, Subash Babu, Vasanthapuram Kumaraswami, Thomas B. Nutman

**Affiliations:** 1 Helminth Immunology Section, Laboratory of Parasitic Diseases, National Institute of Allergy and Infectious Diseases (NIAID), National Institutes of Health (NIH), Bethesda, Maryland, United States of America; 2 National Institute for Research in Tuberculosis, Chetpet, Chennai, India; 3 Biostatistics Research Branch, Division of Clinical Research, NIAID, NIH, Bethesda, Maryland, United States of America; 4 NIAID/NIH-NIRT International Center for Excellence in Research Chetpet, Chennai, India; Fundació Institut d’Investigació en Ciències de la Salut Germans Trias i Pujol, Universitat Autònoma de Barcelona, CIBERES, Spain

## Abstract

**Background:**

Filarial (and other helminth) infections are known to modulate mycobacteria-specific pro-inflammatory cytokine responses necessary for maintaining latency in tuberculosis (TB). We sought to address whether helminth co-infection alters progression to active pulmonary TB in a co-endemic area of South India.

**Methods/Principal Findings:**

Incidence of active pulmonary TB was assessed in 5096 subjects from five villages among helminth-infected (hel^+^) and –uninfected (hel^−^) groups. Baseline stool examinations, circulating filarial antigen, and tuberculin skin testing (PPD) were performed along with chest radiographs, sputum microscopy, and culture. During three follow-up visits each 2.5 years, patients were assessed using PPD tests and questionnaires and—for those with potential symptoms of TB—sputum microscopy and culture. Of the 5096 subjects, 1923 were found to be hel^+^ and 3173 were hel^−^. Follow up interval stool examination could not be performed. In each group, 21 developed active TB over the course of the study. After adjusting for sex, age, BCG vaccination status, and PPD positivity, no difference was seen in active TB incidence between hel^+^ and hel^−^ groups either at baseline (relative risk (RR) 1.60; 95% confidence interval (CI): 0.69, 3.71, *P* = 0·27), or when followed prospectively (RR 1.24; 95% CI: 0.48, 3.18, *P* = 0·66).

**Conclusions/Significance:**

Our findings suggest that, despite the immunomodulatory effects of helminth infection, baseline co-morbid infection with these parasites had little effect on the clinical progression from latent to active pulmonary TB.

## Introduction

*Mycobacterium tuberculosis* (Mtb) infection remains a disease of significant public health importance, particularly in resource-limited parts of the world. According to WHO reports, 1·4 million deaths were associated with tuberculosis (TB) in 2010 worldwide.[1], [2] The 2010 worldwide tuberculosis (TB) incidence was 8·8 million (128 cases/100 000 inhabitants), of which 59% were detected in Asia. Intestinal and tissue-invasive helminth infections geographically overlap with *Mycobacterium tuberculosis* (Mtb) and, because of their chronic nature, induce significant immune-mediated modulation. It is well known that bacillus Calmette-Guérin (BCG) vaccination has been ineffective in preventing adult-onset TB in many parts of the world where helminths are commonplace [3], [4].

Although there are clear risk factors such as HIV infection, immunosuppression, malnutrition, and diabetes predisposing to development of active TB, few studies have addressed the effects of co-incident helminth infection on incident TB, mostly because of the logistic constraints of following large populations longitudinally.

Previous studies examining the interaction between helminth infection and TB have been largely cross-sectional and have produced conflicting results that might, in part, be skewed due to either geographic differences in helminth species prevalence or to the limitations of observational cross-sectional study design. In hospital-based studies from Brazil and Ethiopia, helminth-infected (hel^+^) subjects had a higher prevalence of active TB than did those who were helminth-uninfected (hel^−^), with the most prevalent helminth co-infections being *Ascaris lumbricoides* [5] and *Strongyloides stercoralis*, respectively [6].

To our knowledge, only one study has prospectively examined rates of development of active TB, this in a HIV-hel^+^ cohort from Uganda [7]. Using strict definitions of Mtb infections (culture positivity), this study demonstrated an association between having *Schistosoma mansoni* infection and developing active TB but did not show such an association between the presence of intestinal helminth infection and incident rates of TB.

It is generally accepted that Th1 responses (most notably interferon (IFN)-γ) are required for optimal TB control. Chronic helminth infections—dominated by expansion of both Th2 cells and regulatory T cells (Tregs) producing IL-10 and/or TGF-β [8]—can potentially modulate the Th1-type response necessary to control TB. Thus, to better understand the effect of concurrent helminth infection on developing active TB, we re-examined data obtained previously[9] and additionally followed this cohort of patients in South India prospectively over an 8-year period to assess the role of coincident helminth infection on the incidence and severity of active pulmonary TB.

## Methods

### Ethics Statement

The study was approved by the NIAID Institutional Review Board (protocol number 01-I-N261) as well as the Institutional Review Board of the NIRT (formerly Tuberculosis Research Center). Informed written consent was obtained from subjects enrolled. All data obtained were analyzed anonymously. Parents/legal guardians provided written consent for any subject under the age of 18 years.

### Study Population

The target population included all persons 6–65 years of age from five villages of the Tiruvallur District, Tamil Nadu, located 40 km from Chennai, India. The study population comprised those eligible persons (7397) who consented to study participation. Recruitment was conducted from June 1999 through April 2000, and 5096 persons were enrolled initially as part of the registered study (NCT00342017). Each study participant received a complete medical evaluation including detailed medical history and physical examination. Standardized forms were used for demographic, clinical, and epidemiologic information. BCG vaccination status was assessed by examination of subjects' arms for the characteristic BCG scar.

### Baseline Assessments

We briefly describe the baseline assessments; baseline analysis of this study has been previously reported [9], although with some additional information on active TB rates. All study subjects had one stool examination for parasitic ova and larvae. Whole blood (1 ml) was obtained for determination of circulating filarial antigen (CFA) levels. In addition, 4463 of 5096 (87.6%) subjects were tested (at baseline) for skin-test reactivity to intradermal PPD as described previously [9]. Any person with pulmonary symptoms (cough, fever, chest pain, hemoptysis) had a chest X-ray as well as sputum collection for microscopy and culture for Mtb. Ziehl-Neelsen staining was used to detect the presence of acid-fast bacillus on microscopy, followed by fluorescence microscopy and culture using a modified Petroff method for culture of Mtb [10]. This method used Lowenstein-Jensen media without potato starch. Cultures were examined weekly for 8–9 weeks and were reported as negative if no growth was present by that time.

### Follow-up Assessments

Follow-up assessments were performed at 2- to 2.5-year intervals for a total of three follow-up assessments after the initial assessment. At each follow-up visit, study subjects with symptoms suggestive of active pulmonary TB underwent sputum microscopy, culture, and chest X-ray. Patients with smear positivity and or culture positive for Mtb were defined as active pulmonary TB cases and were referred for definitive antituberculous directly observed chemotherapy through the National (India) tuberculosis program. Study subjects with no active pulmonary TB symptoms and negative tests were considered to be negative for active pulmonary TB. Follow-up stool testing could not be performed. Due to a concern about mass drug administration (MDA) for helminth infections in this area of Tamil Nadu during the entire study period, the entire state was precluded from either Diethycarbamazine (DEC) or albendazole based therapy (either for intestinal helminths) or lymphatic filariasis.

### Statistical Analyses

To assess association of TB with helminth infection at baseline, we first performed a Fisher's exact test. Then, to adjust for other variables, we performed a series of binary generalized linear models (GLMs) using a log link to interpret the results as relative risks [11]. We used age as a continuous variable in the model, as nonparametric estimates of the rates of active TB by age using a 10-year bandwidth showed an approximately linear increase with age. For prospective analyses, we used the total count of active TB incidents with a GLM with Poisson error and a log link, with an offset for years at risk (years from baseline evaluation until the last completed evaluation).

For time-to-first-event analysis, data were interval censored; we used a nonparametric maximum likelihood estimator of the cumulative probability of developing active TB, with a modified bootstrap confidence interval (CI) (using 2000 replications) and the default logrank test in the interval R package (version 1.1–0.0) [12]. Power calculations in the discussion are based on the GLMs using normality assumptions on the parameter estimates, assuming similar PPD positivity rates and loss to follow-up in similar future studies. Analyses used R 3.0.1 [12].

## Results

At the initial visit, 5096 subjects were screened; baseline data are given in Table 1. Although much of the demographic and epidemiologic data for most of this population has been previously reported [9], here we include subjects in whom tuberculin skin testing was missing (or equivocal). Of the 5096 subjects assessed, there were 25 (0.5%) subjects with active TB at baseline and 465 (9%) subjects with active filarial infection based on the presence of CFA. Hookworm infection was found to be the most prevalent intestinal helminth infection and was found in 1524 (30%) subjects. Twenty-seven percent (n = 1384) of the subjects had been BCG vaccinated, and 2400 of the 4463 tested (54%) were tuberculin skin-test positive.

**Table 1:** Baseline Characteristics of Study Population (n = 5096).

Characteristic	%	(n)
Female	54.0	(2753)
Hookworm infection	29.9	(1524)
Circulating filarial antigen positive	9.1	(465)
Bacillus Calmette-Guérin vaccinated	27.2	(1384)
PPD positivity	53.8^a^	(2400)
Culture-confirmed tuberculosis	0.5	(25)

a 663/5096 subjects missing tuberculin skin testing.

At baseline, 12/1923 (0.62%) in the hel^+^ group and 13/3173 (0.41%) in the hel^−^ group (*P* = 0·31) had active pulmonary TB. Among those with active TB, there was little difference between the hel^+^ and the hel^−^ groups in terms of age, gender, BCG, and PPD positivity (Table 2). When we tested for the effect of helminth infection on active TB at baseline by fitting a binary GLM (Table 3), there was no significant association of helminth infection at baseline with the presence of active TB (RR: 1.60; 95% CI 0.69, 3.71; *P* = 0·27) even after controlling for age, gender, BCG vaccination status, and skin test reactivity. There were significant associations with gender (males have a higher risk than females: RR = 6.8, *P* = 0.002) and age (for each decade older, the risk increases by a factor of 1.9, *P*<0·001), and there was a borderline significant association with tuberculin skin test positivity (RR = 6.84, *P* = 0·06). If we perform a similar analysis without including skin test positivity—so that all 5096 subjects can be included—similar results (RR for any helminth infection: 1.51; 95% CI 0.68, 3.30, *P* = 0·30) are obtained. Hookworm or patent filarial infection (CFA-positive subjects) showed no association with having active TB in a model that adjusts for gender, age, BCG vaccination status, and skin test positivity (hookworm RR = 1.54; 95% CI 0.63, 3.56; *P* = 0·32; CFA RR = 0.85; 95% CI 0.14, 2.87; *P* = 0·82).

**Table 2:** Characteristics of Patients with Active TB at Baseline and on Prospective Assessment.

	Active TB at Baseline		Active TB Detected Prospectively	
Characteristic	Helminth-Uninfected	Helminth Infected	Helminth-Uninfected	Helminth-Infected
n	13	12	9	9
Median Age (Range)	51 (23–65)	44.5 (21–65)	52 (35–55)	52 (32–58)
Female: n (%)	2 (15.4)	2 (16.7)	5 (55.6)	2 (22.2)
BCG a scar: n (%)	5 (38.5)	5 (41.7)	2 (22.2)	4 (44.4)
PPD >12 mm: n (%)	10 (90.9)	11 (100)	9 (100)	7 (87.5)
Hookworm: n (%)	NA	10 (83.3)	NA	9 (100)
LF: b n (%)	NA	2 (16.7)	NA	1 (11.1)
Coinfection: c n (%)	NA	0 (0)	NA	1 (11.1)

a Bacillus Calmette-Guérin.

b Lymphatic filariasis (circulating filaria antigen).

c LF and hookworm.

**Table 3:** Results Using the Generalized Linear Model for Baseline Data.

Characteristic	Relative Risk (RR)	Confidence Limit		Two-sided *P*-value
		Lower 95%	Upper 95%	
Age (RR for 10 years older)	1.91	1.36	2.81	*P*<0.001
Sex (RR for males compared with females)	1.63	0.64	4.45	*P* = 0.316
BCG a	0.98	0.34	2.54	*P* = 0.964
Any helminth infection	1.24	0.48	3.18	*P* = 0.656
PPD positivity	6.52	1.3	118.5	*P* = 0.071

a Bacillus Calmette-Guérin.

**Figure 1:**
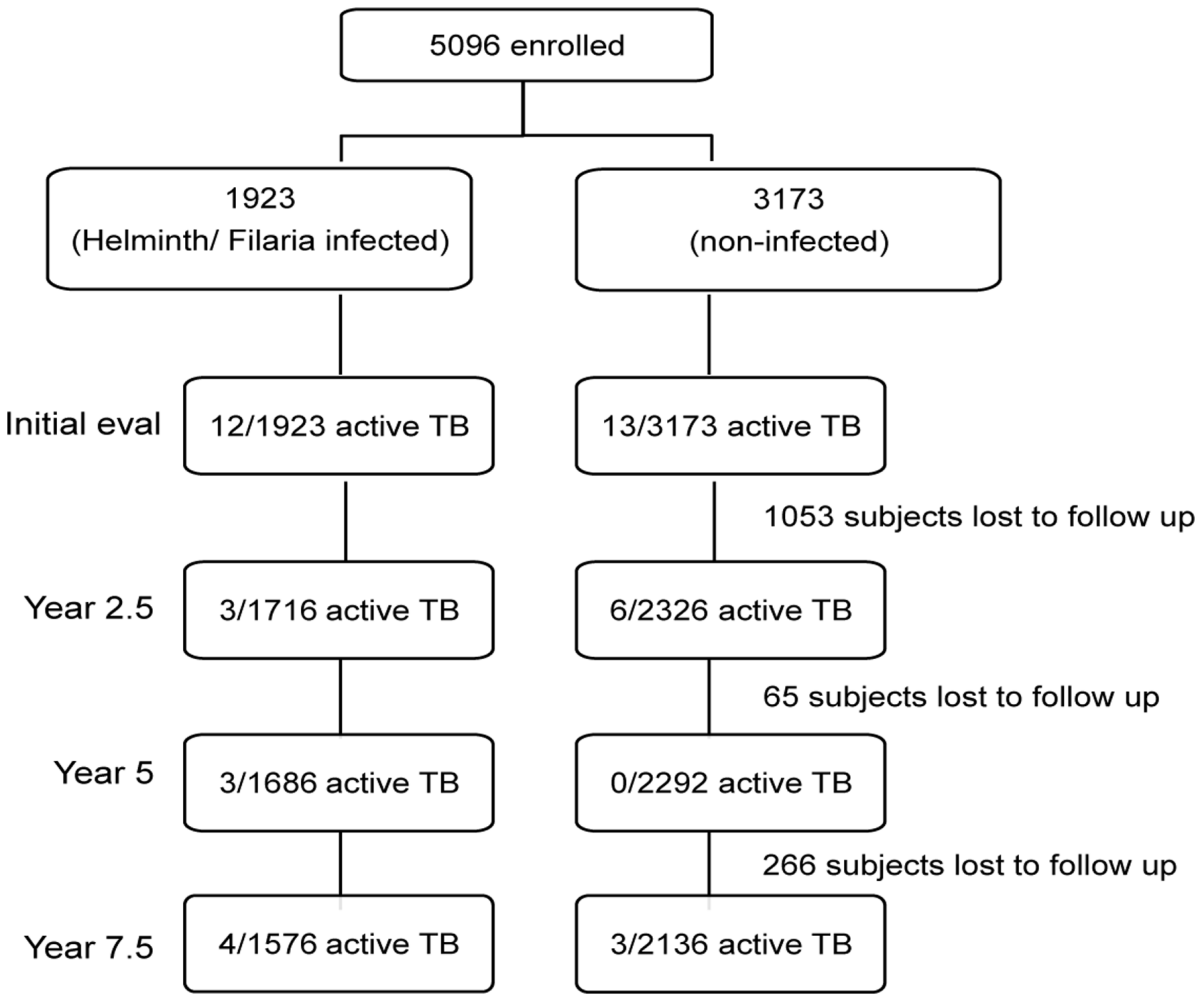
Flow diagram showing the numbers of subjects with active TB and total number evaluated, along with loss-to-follow-up at each evaluation time point of the study.

We followed most of the subjects for approximately 7.5 years, with TB evaluations every 2–2.5 years. We had prospective data on 4043 subjects for a total of 28 928 person-years with a median follow-up of 7.4 years. Nearly all of the missing subjects were from a single village (n = 1047) that collectively decided not to participate after the baseline evaluation. There were 18 subjects who developed active TB during the prospective portion of the study (Figure 1). Sixteen of these 18 subjects had a single episode of active TB, whereas there was one subject who had had active TB at baseline and one who relapsed after treatment of his first episode of active TB during the 7.5 years of follow-up. In Table 2, we give descriptive statistics on age, gender, BCG vaccination, and tuberculin positivity of those with active TB, stratified by those with and those without helminth infection at baseline. We next tested for differences between the variables through interaction tests on the Poisson GLM. The crude rates of active TB were 10/12 279 = 81.4 per 100,000 person-years for the hel^+^ group and 9/16 649 = 54.1 per 100,000 persons years for the hel^−^ group (RR = 1.51; 95% CI 0.61, 3.79; *P* = 0.37).

Controlling for age, gender, BCG vaccination status, and PPD positivity, we still found no significant effect of helminth infection on the incident rates of active TB infection (from GLM results of Table 4 on n = 3570 subjects: RR = 1.24; 95% CI 0.48, 3.18; *P* = 0.66). There were significant effects for age (RR for each decade older = 1.91; 95% 1.36, 2.81; *P*<0.001) and a trend toward significance for tuberculin skin test positivity (RR = 6.52; 95% CI 1.30, 118.5; *P* = 0·07). In this model, we included no interaction terms because when we test for interactions of baseline helminth infection with any of the four variables (age, gender, BCG vaccination, PPD positivity) at once, we found no significant effects (overall analysis of deviance test, *P* = 0.15). Results were similar to those seen with helminth infection if we omit the presence of tuberculin skin test positivity to include more subjects (n = 4043; RR for any baseline helminth infection  = 1.38; 95% CI 0.55, 3.48; *P* = 0·49).

**Table 4:** Results Using the Generalized Linear Model for Prospective Data.

Characteristic	Relative Risk (RR)	Confidence Limit		Two-sided *P*-value
		Lower 95%	Upper 95%	
Age (RR for 10 years older)	1.91	1.36	2.81	*P*<0.001
Sex (RR for males compared with females)	1.63	0.64	4.45	*P* = 0.316
BCG a	0.98	0.34	2.54	*P* = 0.964
Any helminth infection	1.24	0.48	3.18	*P* = 0.656
PPD positivity	6.52	1.3	118.5	*P* = 0.071

a Bacillus Calmette-Guérin.

**Figure 2:**
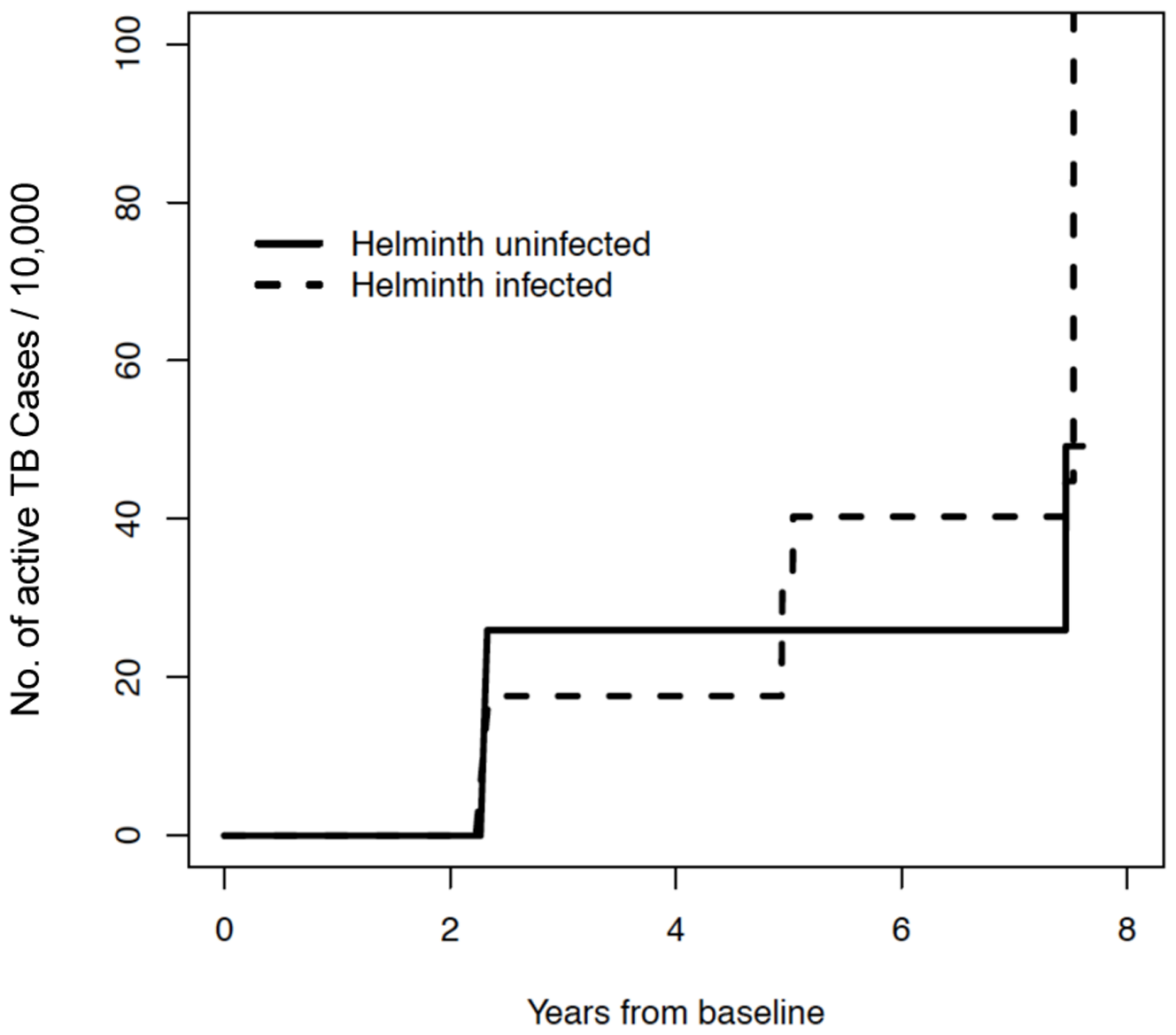
Time to development of active TB (in years) in helminth-infected (dotted line) and helminth-uninfected (solid line) subjects using nonparametric maximum likelihood estimates.

Note that there was one subject who relapsed and therefore contributed two incidents to the prospective analysis. An alternative analysis would be one that examined time-to-first-episode of active TB. The estimate of the cumulative proportion is given in Figure 2. A logrank test shows no significant difference between hel+ and hel^−^ subjects (*P* = 0.49).

**Figure 3:**
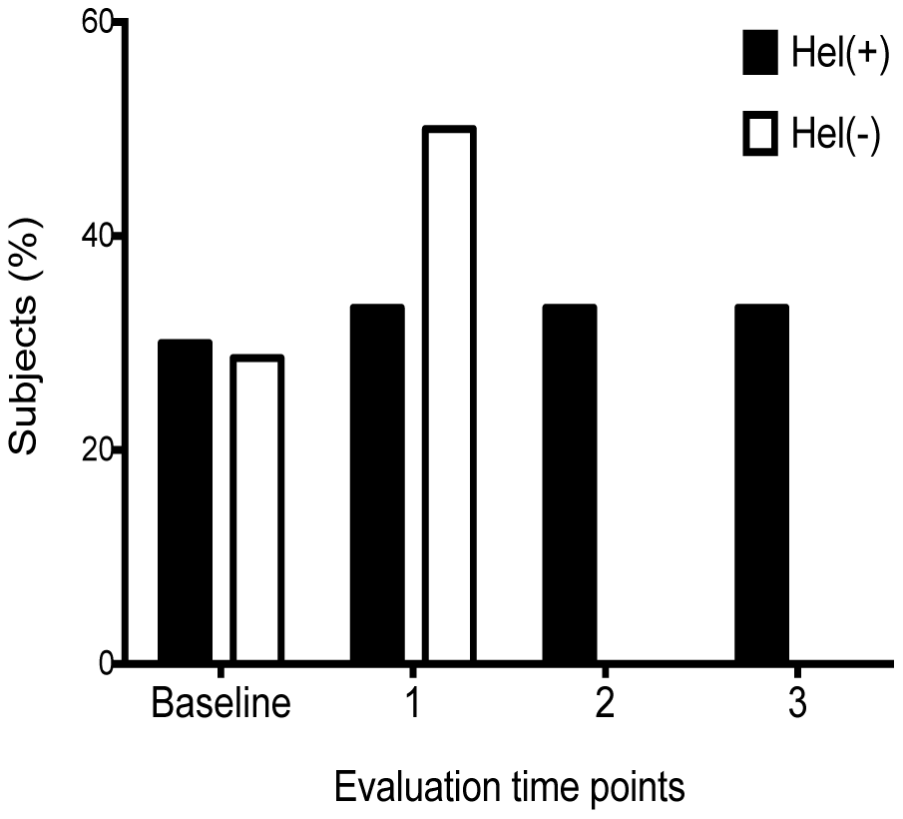
Severity of disease among subjects with active TB. Subjects in each group with bilateral lesions on chest radiographs (expressed as a percentage of all subjects with active TB who had complete chest X-ray data was available at the particular time point of evaluation) are shown for helminth-infected (dark bars) and helminth-uninfected (open bars) at each assessment time point. The percentage of subjects with evidence of severe disease in the hel^+^ and hel– groups, respectively, were: 3/10 (30%) and 2/7 (29%) at baseline; 1/3 (33%) and 3/6 (50%) at 2.5-year follow-up; 1/3 (33%) and 0 at 5-year follow-up; and 1/3 (33%) and 0/1 at 7·5-year follow-up with no significant differences seen between groups overall or at each time point of evaluation.

Previous studies [7], [13] have suggested that the presence of intestinal helminths may have an impact on the severity of mycobacterial disease. We therefore assessed the percentage of subjects in each group who developed severe pulmonary disease based on chest X-ray evaluation (either at baseline or during the prospective part of the study). For all patients who had chest X-ray data available, severe disease was defined as evidence of bilateral disease consistent with active TB (subsequently confirmed by sputum microscopy and/or culture for the presence of acid-fast bacillus). Sputum smear grade (data not shown) analysis revealed high-grade sputum positivity (2+) in three subjects in the hel^+^ group and two subjects in the hel^−^ group. There was no difference overall in the degree of sputum positivity or in the number of subjects with smear-negative, culture-positive TB between the two groups. This assessment of severity was possible only on subjects in whom complete chest X-ray data was available at the particular time point of evaluation. As shown in Figure 3, the percentage of subjects with evidence of severe disease in the hel^+^ and hel– groups, respectively, were: 3/10 (30%) and 2/7 (29%) at baseline; 1/3 (33%) and 3/6 (50%) at 2.5-year follow-up; 1/3 (33%) and 0 at 5-year follow-up; and 1/3 (33%) and 0/1 at 7·5-year follow-up, and there were no significant differences between groups at each evaluation time point (*P* = 1, Fisher's exact test) and overall (*P* = 1.0, Fisher's exact test).

## Discussion

Our study assessed in both a cross-sectional and a longitudinal prospective manner the incidence of active pulmonary TB in a group of subjects in a well-delimited geographic area with a high prevalence of coincident helminth infections. Subjects were from broadly similar socio-demographic backgrounds, and stringent criteria were used for the diagnosis of active pulmonary TB. In 1999 Directly Observed Treatment, Shortcourse (DOTS) strategy was implemented in the area of study after which the rates of culture-positive and smear-positive pulmonary tuberculosis decreased by 11.9 and 5.6% respectively in the first 2.5 years [14] Thereafter, there has been a decrease of incident TB cases in this area with rates of approximately 3-4 cases/1000 reported for every 2.5 year follow-up period. These reported rates are also consistent with incident TB cases reported in Tamil Nadu overall.[15]. We were also able to limit loss-to-follow-up—a problem that remains a challenge of studies performed in resource-limited settings—on serial assessments.

Our study demonstrates that helminth infection (either soil-transmitted helminths or *W. bancrofti*) at initial assessment, did not substantially increase the incidence of active pulmonary TB in this population. Hookworm infection was the most prevalent soil transmitted helminth infection in our study as has been shown by other groups[16]. We were, unfortunately, not able to collect multiple stool samples from study subjects which might have given an improved estimate of the prevalence of these infections as has been shown in other surveys [16]–[18]No difference in tuberculin skin test reactivity or in BCG vaccination status was seen between hel^+^ and hel^–^ groups [9]. There was also no significant difference in time to development of active TB or in severity of disease between the groups. Ours is the first large-scale prospective study addressing active TB incidence in a helminth-infected population.

*In vivo* rodent helminth/Mycobacteria co-infection models have failed to provide a consensus on the impact of these worm infections on the outcome of mycobacterial infections. Studies in mice showed that the presence of the intestinal helminth *Nippostrongylus brasiliensis* had no effect on *Mycobacterium bovis* bacillary load at either 4 or 12 weeks post infection compared with uninfected controls [19]. A Toxocara model with Mtb infection showed no differences in the IFN-γ response, bacterial loads, T cell proliferation, or histopathologic changes despite there being an alteration in the composition of the alveolar infiltrate[2]. More recently, cotton rats co-infected with the rodent filarial parasite *Litomosoides sigmodontis* and Mtb [20] showed no alteration in antigen-specific IFN-γ responses, PPD-specific T cell proliferation, or tissue bacillary loads of Mycobacteria in Mtb-infected rats. These studies are in contrast to studies in *Schistosoma mansoni* infection that have demonstrated impaired mycobacterial clearance in the lungs of coinfected mice [21].

Our study also did not show any difference in the time to active TB in this cohort, suggesting that although helminth-induced immunomodulation can alter responses to bystander antigens [22], [23], overall susceptibility to active TB may not differ substantially in the presence or absence of these helminth infections.

The human host response to TB is still being characterized, and—although there are some clearly defined examples of immune modulation leading to active disease (i.e., use of anti-TNF antibodies, advanced HIV, and older age)—the clinical effects of immune deviation as a consequence of concurrent helminth infection have not been fully elucidated. It has been postulated that, in addition to bacterial load, the early innate response as well as subsequent T cell-mediated adaptive responses may be involved in a protective immune response to Mtb [24]. Data from human genetic studies and animal models of vaccine-induced protection of active TB have also demonstrated the central protective role of IFN-γ [25]–[27].

Helminth infections, by their nature, lead to potent induction of Th2 responses (acutely) characterized by increased IL-4, IL-5, and IL-13 and—over time—to an expansion of Tregs [28]. These factors profoundly impair the Th1 response to PPD and mycobacterial culture filtrate protein in humans [29], [30] and in animal models [21], [31], [32] with a return toward normal of Mtb-specific Th1 responses following definitive treatment of the helminth infection [33]. It has also been demonstrated previously that upregulation of CTLA-4 and PD-1 [34] leads to diminished Th1 and Th17 responses in Fil^+^ patients with latent TB. Blockade of these molecules led to reversal of the downregulated responses. Similar findings have been recently reported in a hookworm/Mtb study [23]. In addition, there is accumulating evidence that helminth infection may be associated with generation of alternatively activated macrophages (M2) that might have impaired machinery to respond to bacterial infections [35]. Indeed, in a mouse co-infection model of *Nippostrongylus brasiliensis* and Mtb, impaired immune defense against TB was dependent on IL-4 generation and subsequent alternative pathway activation [36].

Finally, we saw no differences in overall extent and severity of pulmonary TB disease based on chest radiography. Using a standardized and validated scoring system [10], we did not note any differences in the degree of cavitary disease between the hel^+^ and hel^−^ groups (data not shown). Using bilateral disease as a simplified measure of increased severity, we noted no significant differences between the hel^+^ and hel^−^ groups. In a *Strongyloides*/Mtb co-infection study, similar scoring systems [37], [38] showed no significant increase in the frequency of cavitary disease [39]. In the *Strongyloides*-infected group, the number of disease-involved lung zones was significantly higher at the end of TB treatment.

Sputum-smear grading at the time of diagnosis in immunocompetent adults has been shown previously to correlate with culture conversion [40] and has also been shown to correlate with severity of disease by chest radiography [37]. Our study was unable to find any significant differences in the degree of sputum-smear positivity between groups.

One of the limitations of our study was our inability to perform serial interval evaluations for intestinal and tissue invasive helminth infections over the course of follow-up as it is well known that endemic populations have high rates of reinfection [41].Helminth infection status, as assessed at baseline is, however, an important predictor of immune responses to bystander antigen responses[23], [34] and the reversal of these immunoregulatory effects has often required chronic and repeated drug therapy and no reinfection [42]. Concern about implementation of MDA in this area by the government of Tamil Nadu precluded systematic treatment with albendazole for intestinal helminth and/or filarial disease. Therefore, the helminth infection status was relatively stable throughout the period of assessment. All subjects were eventually treated after the MDA program was rolled out after 2007 in the study area [43]. Subjects were also not tested for HIV status but the prevalence of HIV among subjects with tuberculosis is <1% in this area[14] and therefore less likely to be a significant confounder. Despite these limitations, we believe our study provides important information on incident TB rates between groups with or without helminth infection at baseline.

Another potential bias in our study is the lack of systematic assessment of socioeconomic factors as well as household overcrowding and exposure to cigarette and indoor smoke—known risk factors for developing active TB in resource-limited parts of the world [44]. It would, however, be safe to assume that the majority of this population was from the same socioeconomic strata, with similar living conditions, as has been reported previously [9]. We were also unable to do mycobacterial strain typing, which might have provided information as to how many infection clusters in individual villages were due to activation of latent TB or were newly acquired [45]. This study does, however, provide powerful evidence that immune deviation caused by hookworm infection, although highly prevalent, might not lead to a higher incidence of active pulmonary TB in a co-infected cohort in endemic settings. If the effects in this study hold, further large population-based studies would need sample sizes about three times larger (about n = 15 000) for cross-sectional studies and about 8 to 20 times larger (200 000 to 500 000 person-years) for prospective studies to show significant associations of helminth infection on active TB incidence.
